# Normalization of systemic arterial hypertension following removal of posterior fossa hemangioblastoma: a case report

**DOI:** 10.1186/1757-1626-2-7106

**Published:** 2009-04-29

**Authors:** Hooshang Saberi, Ali Tayebi Meybodi, Mehdi Zeinalizadeh

**Affiliations:** 1 Departement of Neurosurgery, Brain and Spinal Injuries Repair Research Center (BASIR), Imam Khomeini Hospital, Tehran University of Medical Sciences, Tehran, 14197, Iran; 2 Departement of Neurosurgery, Imam Khomeini Hospital, Tehran University of Medical Sciences, Tehran, 14197, Iran

## Abstract

The concept of compression of the rostral ventrolateral medulla as a cause for hypertension is gaining more and more interest. This report is about a 36-year-old male with a three years history of hypertension who presented with a posterior fossa mass suggestive of a hemangioblastoma. Laboratory and imaging studies ruled out the presence of von Hippel-Lindau disease and/or concomitant pheochromocytoma. Post-surgical blood pressure monitoring revealed a 40 mmHg decline in blood pressure. It could be hypothesized that alleviation the compressive effect of the tumour on the rostral ventrolateral medulla as proposed by previous studies could be a contributing factor.

## Introduction

Hemangioblastomas (Grade I in World Health Organization classification of brain tumors) constitute around 2-3% of all primary nervous system tumors and although they can occur at all sites from the cerebral cortex to peripheral nerves, are most commonly found in the cerebellar hemispheres [1,2]. Presentations are related to mass effects on the neural structures and raised intracranial pressure. Various abnormalities such as retinal hemangioblastomas, renal and pancreatic cysts, renal carcinoma, pheochromocytoma, pancreatic islet cell tumors, endolymphatic sac tumors, and papillary cystadenoma of the epididymis are reported in 25% of hemangioblastomas as a part of von Hippel-Lindau disease (VHL) [1,3]. However, systemic hypertension not associated with pheochromocytomas in sporadic cases of hemangioblastoma is a rarity. The intriguing association between arterial hypertension and posterior fossa tumors and its amelioration after total surgical removal has been touched in this case.

## Case presentation

**Figure 1:**
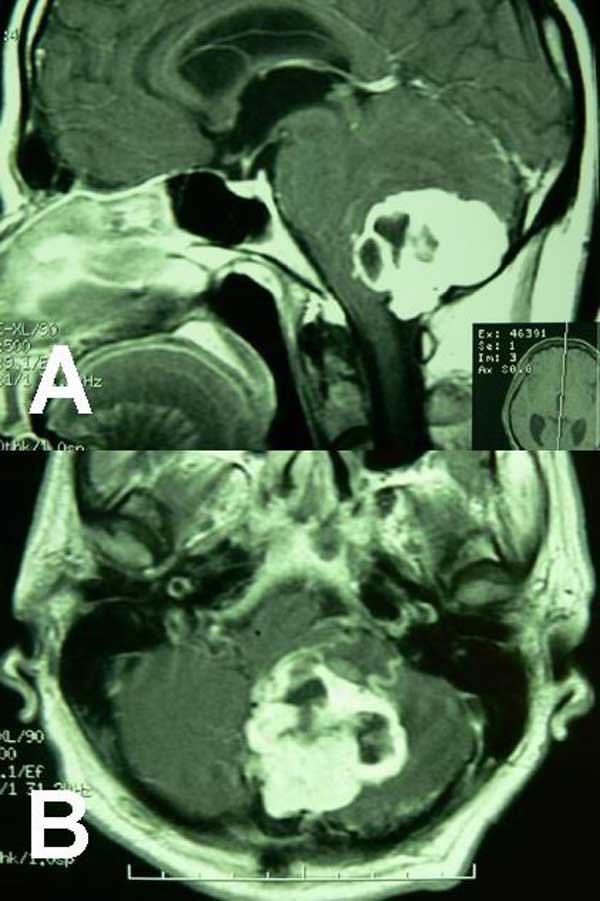
**(A) and (B) Sagittal and axial T1-weighted magnetic resonance images of brain after contrast injection showing a solid-cystic densely enhancing mass in the left cerebellar hemisphere with compressive effect upon the fourth ventricle causing obstructive hydrocephalus**.

**Figure 2:**
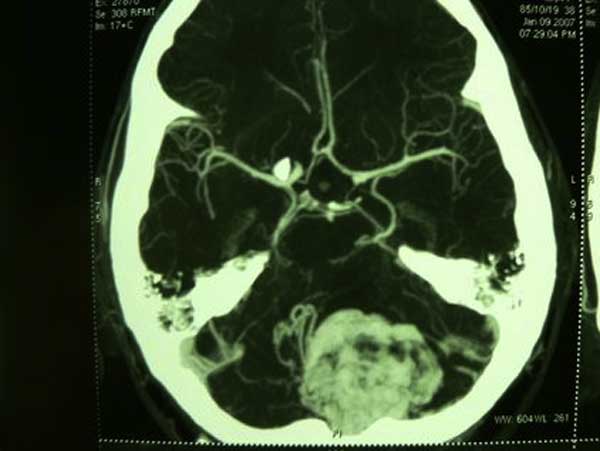
**Computed tomographic angiogram of brain showing a highly vascular mass supplied by multiple tortuous feeders originating from cerebellar cortex as well as postero-inferior cerebellar artery**.

A 36-year-old Iranian male with Caucasian ethnicity referred to the emergency department of our institute with a chief complaint of severe suboccipital headache, vomiting and frequent drop attacks. He had arterial hypertension for 3 years and was taking atenolol 50 mg/day. Family history was negative for secondary causes of arterial hypertension including adrenal disease. Physical examination revealed blood pressure of 180/100 mmHg, bilateral papilledema and truncal ataxia with gaze-dependent horizontal nystagmus. Lab tests showed polycythemia (Hemoglobin = 18 g/dL). Contrast enhanced brain MRI showed a round extra-axial heterogeneous densely enhanced 4 × 4 cm mass with irregular margins. It was located in the left cerebellar hemisphere, posterolaterally obliterating the fourth ventricle, causing non-communicating hydrocephalus. The mass encroached onto the cavity of the fourth ventricle anterolaterally, abutting the right half of the brainstem (Figure 1). Magnetic resonance imaging of the whole spine was negative for spinal lesions. Regarding severe headache and vomiting due to the non-communicating hydrocephalus, a ventriculoperitoneal shunt was inserted on an emergency basis, postponing tumor removal for further evaluation. However, the patient remained hypertensive despite alleviated symptoms of raised intracranial pressure. Computed tomographic (CT) angiography (with 3D reconstruction) revealed a highly vascular lesion irrigated by hypertrophied right posterior inferior cerebellar artery (PICA) (Figure 2). Abdominal CT scan showed 2 simple cortical cysts in the right kidney. Urinary 24-hour metanephrine and vanillylmandelic acid (VMA) excretion were found to be within normal limits. Ophthalmology consult did not reveal retinal vascular lesions.

**Figure 3:**
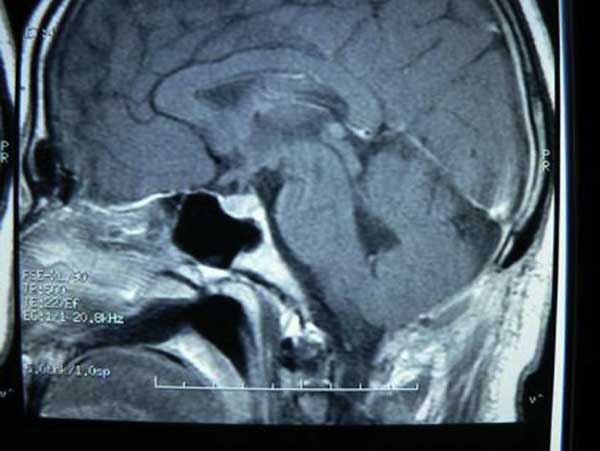
**Post-operative magnetic resonance image of brain revealing no residual tumor and restoration of the 4^th^ ventricular contour**.

A midline posterior fossa craniectomy was performed. Following durotomy, the mass appeared as a reddish lobulated tumor with multiple feeders from cerebellar cortex and the bridging PICA which were coagulated and divided. Not surpassing the surrounding gliotic border, the tumor was totally resected. Post-operative period was uneventful and the obtained MRI revealed no residual tumor (Figure 3). Surprisingly, blood pressure showed a drastic decline of about 40 mmHg and the patient remained normotensive for the next 6 months of follow-up, needing no anti-hypertensive medication. The pathological examination confirmed the diagnosis of hemangioblastoma. The specimen stained positive for Glial Fibrillary Acidic Protein (scattered), S100 (scattered), MIB1 and CD34 and negative for Cytokeratin, epithelial membrane antigen (EMA), Neuron Specific Enolase (NSE) and neuropeptide Y.

## Discussion

Secondary hypertension may be caused by a myriad of secretory disorders, including some tumors such as pheochromocytoma and aldostrone-secreting masses. However, not all these hypertensive states could be attributed to humoral factors. In 1979, Janneta and Gendell introduced the concept of neurogenic hypertension caused by pulsatile vascular compression of the left lateral medulla [4]. Animal studies using various models have confirmed the presence of a subpial neuronal group (C-1) in the rostral ventrolateral medulla (RVLM) producing a transient pressor response when stimulated chemically, electrically or mechanically [5]-[8]. Likewise, there has been an abundance of evidence based upon experimental studies, postmortem anatomic dissections, and radiographic findings to portend that compression of the medulla elicits sympathetically mediated hypertension, even non-pulsatile and regardless of laterality [9]. As a therapeutic intervention, many reports have been published in which the microvascular decompression of the RVLM is associated with a decline in blood pressure of hypertensive patients [4,10]. Also, there have been two reports of resolution of hypertension after posterior fossa decompression for Chiari malformation type I in which the compressive effect of the herniated cerebellar tonsils on the cervicomedullary junction seemed to be the core pathophysiologic process [11,12]. To date, only few cases of arterial hypertension have been published, in which the associated hypertension (HTN) resolved following tumor resection [13]. In the first of such reports, Cameron and Doig reported two cases of cerebellar tumors (hemangioblastoma) with brainstem compression presenting with malignant hypertension [14]. Hedderwick et al. reported a case of hemangioblastoma of the cervicomedullary junction, containing neuropeptide Y in which the associated hypertension abated after tumor resection [15]. However, this marker was not positive in our patient. Approximately 25% of hemangioblastomas are associated with VHL [1]. Our patient did not manifest with retinal and adrenal lesions neither had he high urinary cathecholamine excretion precluding the diagnosis of VHL and possible secreting function of the tumor. The older age, and the solitary tumor of cerebellum, along with negative family history for autosomal dominant VHL, all were in favor of the diagnosis of sporadic posterior fossa hemangioblastoma.

Mitigation of arterial hypertension after resection of the tumor in our patient, as well as negative laboratory and imaging studies for secondary causes for HTN supports the proposed mechanism of RVLM compression, as the pathogenesis of HTN.

## Conclusion

There had been reports of posterior fossa tumors associated with arterial hypertension. The plausible mechanism of rostral ventrolateral medullary compression is gaining interest as a cause for hypertension. The present case is another clue advocating the proposed mechanism.

## List of abbreviations

VHL: von Hippel-Lindau disease; MRI: Magnetic resonance imaging; CT: Computed Tomography; PICA: Posterior inferior cerebellar artery; VMA: Vanillylmandelic acid; EMA: Epithelial membrane antigen; NSE: Neuron Specific Enolase; RVLM: Rostral ventrolateral medulla; HTN: Hypertension.

## Consent

Written informed consent was obtained from the patient for publication of this case report and accompanying images. A copy of the written consent is available for review by the Editor-in-Chief of this journal.

## Competing interests

The authors declare that they have no competing interests.

## Authors' contributions

The authors contributed to surgery and preparation of the manuscript. The primary draft and its final revision was done by ATM. HS was the attendant surgeon and prepared the primary draft and revised the manuscript. MZ aided in surgery and revised the manuscript.

## References

[B1] Conway JE Chou D Clatterbuck RE Brem H Long DM Rigamonti D Hemangioblastomas of the central nervous system in von Hippel-Lindau syndrome and sporadic disease Neurosurgery 2001 48 55 62 10.1097/00006123-200101000-00009 11152361

[B2] Glasker S van Velthoven V Risk of hemorrhage in hemangioblastomas of the central nervous system Neurosurgery 1995 57 71 76 10.1227/01.NEU.0000163250.71951.18 15987542

[B3] Sano T Horiguchi H von Hippel-Lindau disease Micros Res Technol 2003 60 159 164 10.1002/jemt.10253 12539169

[B4] Jannetta PJ Gendell HM Clinical observations on etiology of essential hypertension Surg Forum 1979 30 431 432 538657

[B5] Dampney RA Goodchild AK Robertson LG Montgomery W Role of ventrolateral medulla in vasomotor regulation: a correlative anatomical and physiological study Brain Res 1982 14 223 235 10.1016/0006-8993(82)90056-7 6128058

[B6] Dormer KJ Bedford TG Cardiovascular control by the rostral ventrolateral medulla in the conscious dog Prog Brain Res 1989 81 265 277 10.1016/S0079-6123(08)62016-6 2616786

[B7] Jannetta PJ Segal R Wolfson SK Jr Dujovny M Semba A Cook EE Neurogenic hypertension: etiology and surgical treatment. II. Observations in an experimental nonhuman primate model Ann Surg 1985 202 253 261 10.1097/00000658-198508000-00018 4015232 PMC1250882

[B8] Ross CA Ruggiero DA Park DH Joh TH Sved AF Fernandez-Pardal J Tonic vasomotor control by the rostral ventrolateral medulla: effect of electrical or chemical stimulation of the area containing C1 adrenaline neurons on arterial pressure, heart rate, and plasma catecholamines and vasopressin J Neurosci 1984 4 474 494 6699683 10.1523/JNEUROSCI.04-02-00474.1984 PMC6564896

[B9] Levy EI Clyde B McLaughlin MR Jannetta PJ Microvascular decompression of the left lateral medulla oblongata for severe refractory neurogenic hypertension Neurosurgery 1998 43 1 6 10.1097/00006123-199807000-00001 9657182

[B10] Nakamura T Osawa M Uchiyama S Iwata M Arterial hypertension in patients with left primary hemifacial spasm is associated with neurovascular compression of the left rostral ventrolateral medulla Eur Neurol 2007 57 150 155 10.1159/000098466 17213721

[B11] Naderi S Acar F Acar G Men S Resolution of neurogenic arterial hypertension after suboccipital decompression for Chiari malformation: Case report J Neurosurg 2005 102 1147 1150 10.3171/jns.2005.102.6.1147 16028778

[B12] Parker EC Teo C Rahman S Brodsky MC Complete resolution of hypertension after decompression of Chiari I malformation Skull Base Surg 2000 10 149 152 10.1055/s-2000-9511 17171139 PMC1656827

[B13] Kan P Couldwell WT Posterior fossa brain tumors and arterial hypertension Neurosurg Rev 2006 29 265 269 10.1007/s10143-006-0036-6 16924459

[B14] Cameron SJ Doig A Cerebellar tumours presenting with clinical features of phaeochromocytoma Lancet 1970 7 492 494 10.1016/S0140-6736(70)91579-5 4190179

[B15] Hedderwick SA Bishop AE Strong AJ Ritter JM Surgical cure of hypertension in a patient with brainstem capillary haemangioblastoma containing neuropeptide Y Postgrad Med J 1995 71 371 372 10.1136/pgmj.71.836.371 7644403 PMC2398139

